# N-Doped Carbon Xerogels as Pt Support for the Electro-Reduction of Oxygen

**DOI:** 10.3390/ma10091092

**Published:** 2017-09-17

**Authors:** Cinthia Alegre, David Sebastián, María E. Gálvez, Estela Baquedano, Rafael Moliner, Antonino S. Aricò, Vincenzo Baglio, María J. Lázaro

**Affiliations:** 1 Instituto de Carboquímica, Consejo Superior de Investigaciones Científicas (CSIC), C/Miguel Luesma Castán 4, 50018 Zaragoza, Spain; cinthia@icb.csic.es (C.A.); estela.baquedano@gmail.com (E.B.); rmoliner@icb.csic.es (R.M.); mlazaro@icb.csic.es (M.J.L.); 2 Istituto di Tecnologie Avanzate per l’Energia “Nicola Giordano”, Consiglio Nazionale delle Ricerche (CNR), Salita Santa Lucia sopra Contesse, 5, 98126 Messina, Italy; arico@itae.cnr.it; 3 Sorbonne Universités, Universite Pierre et Marie Curie (UPMC) Paris, Institut Jean le Rond D’Alembert, UMR CNRS 7190, 78210 Saint-Cyr L’Ecole, France; elena.galvez_parruca@upmc.fr

**Keywords:** N-doped, carbon xerogels, Pt-catalysts, oxygen reduction reaction

## Abstract

Durability and limited catalytic activity are key impediments to the commercialization of polymer electrolyte fuel cells. Carbon materials employed as catalyst support can be doped with different heteroatoms, like nitrogen, to improve both catalytic activity and durability. Carbon xerogels are nanoporous carbons that can be easily synthesized in order to obtain N-doped materials. In the present work, we introduced melamine as a carbon xerogel precursor together with resorcinol for an effective in-situ N doping (3–4 wt % N). Pt nanoparticles were supported on nitrogen-doped carbon xerogels and their activity for the oxygen reduction reaction (ORR) was evaluated in acid media along with their stability. Results provide new evidences of the type of N groups aiding the activity of Pt for the ORR and of a remarkable stability for N-doped carbon-supported Pt catalysts, providing appropriate physico-chemical features.

## 1. Introduction

Fuel cell technologies have received considerable interest in the last decades as energy conversion devices due to their potential to reduce both pollutant emissions and the dependence on fossil fuels [1,2,3,4]. It can be stated that today, fuel cells are already a reality, with the implementation in the automotive business of electric vehicles powered by a H_2_-fed polymer electrolyte membrane fuel cell (PEMFC), Toyota, Hyundai, and Honda are a few examples [5]. Nevertheless, in order to further expand the implementation of fuel cells, there are some issues that still need to be improved, like their low performance, durability, and high cost [5].

To decrease the overall cost of the catalysts, two main strategies are pursued: the use of non-platinum group metal catalysts, usually based on transition-metal-carbon-nitrogen networks [6,7,8] or the use of ultra-low loading of platinum [8,9,10]. Regarding the second strategy, it is widely known that the use of carbonaceous supports for electrocatalysts in PEMFC reduces costs (lower amounts of noble metals are needed) because of the improvement of catalytic activity [5,11,12,13], given that their physico-chemical properties can greatly affect the electrocatalyst characteristics. Currently, carbon blacks are typically employed due to the excellent combination of structural and textural properties. These favor the electronic transport together with a proper dispersion of the noble metal particles, resulting in an enhancement of catalyst performance [14]. However, using carbon nanomaterials such as carbon nanotubes, carbon nanofibers, graphene, and carbon gels, etc., has led to enhanced performances when compared to conventional carbon blacks [5,15,16,17,18].

Carbon gels (aero-, cryo-, and xerogels) produced through a simple sol-gel procedure are nanoporous carbons widely employed in energy-related applications. Among them, carbon xerogels are more easily produced, as there is no need for supercritical nor freeze drying, more complicated, and expensive drying processes. Carbon xerogels possess unique properties (mesoporosity, electrical conductivity, high purity, etc.) that can be further tuned by doping with heteroatoms such as N, B, S, or P [19,20,21]. Doping carbon materials with nitrogen improves both activity and durability of the catalysts, due to both nitrogen functionalities and structural defects created [22,23,24,25,26,27,28,29,30]. The durability of catalysts is an important aspect, since carbon materials undergo strong degradation processes when subjected to highly positive potentials. So it is vital to improve the resistance to oxidation of these materials in order to obtain an active and durable catalyst [31,32,33,34]. N-doped carbon xerogels have been recently studied for different catalytic applications, such as hydrogen adsorption, removal of contaminants, lithium batteries, and catalyst supports, etc. [29,35,36,37,38,39,40,41,42].

In the present work, N-doped carbon xerogels (N-CXGs) were synthesized introducing melamine as gel co-precursor. N-CXGs were used as support for low loading Pt electrocatalysts (Pt/N-CXGs) and evaluated for the electro-reduction of oxygen in acid medium. The stability of the Pt/N-CXGs was investigated by means of an accelerated stress test consisting of potential cycling in order to assess the resistance to degradation of these N-doped carbon-supported catalysts.

## 2. Results

### 2.1. N-Doped Carbon Xerogels

Carbon xerogels (CXGs), both with and without melamine, were prepared with two different resorcinol (R) to sodium carbonate (C) ratios (R/C): 130 and 300. Resorcinol to melamine and resorcinol to formaldehyde ratios, along with the amount of solvent were kept constant, as detailed in the experimental section. CXGs are herein named according to the R/C ratio employed, together with an N prefix to indicate N-doped CXGs (i.e., N-CXG-130 stands for N-doped CXG with R/C = 130). Some textural properties of the CXGs and N-CXGs obtained are presented in Table 1. Bare CXGs (i.e., synthesized without nitrogen precursors) presented high surface areas of 461 and 587 m^2^·g^−1^, with different pore development as evidenced by N_2_ adsorption at partial pressure approaching one. CXG-130 showed a low pore volume of 0.29 cm^3^·g^−1^, mainly attributed to micropores (80%), whereas CXGs synthesized with higher resorcinol/sodium carbonate ratio, CXG-300, developed a porous structure with a larger pore volume (0.65 cm^3^·g^−1^) and a predominance of mesopores (85%). The differences in textural development are caused by the growth of resorcinol-formaldehyde monomers during the gelation process, which is known to be favored at low pH, also increasing the density of mesopores in the CXG [43].

The introduction of melamine in the synthesis of the xerogel has an effect on the textural properties depending on the R/C ratio used. In N-CXG-130, there is an increase of surface area together with a large increase of pore volume when employing melamine. Whereas, a significant decrease of both surface area and pore volume are observed in N-CXG-300. This is due to a higher degree of collapse of the N-doped gel in comparison to the undoped counterpart. Gels containing mixtures of melamine and formaldehyde are more prone to collapse given their higher fragility [44,45]. Pérez-Cadenas et al. found that N-doped xerogels present a narrow microporosity [40]. It appears that a trade-off situation has to be found between the organic gel precursors (resorcinol, melamine) and sodium carbonate for the development of a highly mesoporous structure [30,43,46].

**Figure 1:**
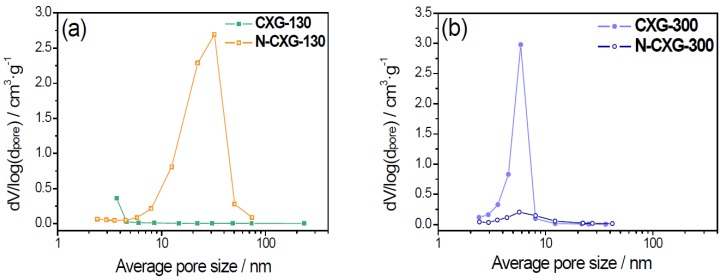
Pore size distribution for N-doped xerogels and their corresponding undoped counterparts (**a**) low R/C and (**b**) high R/C.

Pore size distribution (PSD), as shown in Figure 1, corroborates the different effect of introducing melamine depending on the R/C ratio. When R/C is low, N-doping enhances the development of the porous structure. N-CXG-130 has a PSD centered around 30–40 nm. On the other hand, when R/C is high, the introduction of N leads to the collapse of the porous structure and to a lower pore size, being PSD centered around 5–6 nm for N-CXG-300.

The structure of CXGs was investigated with Raman spectroscopy. The main results, including the ratio between the D and G bands, as well as the position of the latter are also summarized in Table 1. Doping with N decreases the structural order of the synthesized CXGs, as indicated by the shift of G band to higher frequencies and the increase of the I_D_/I_G_ ratio. Resorcinol and melamine compete in the formaldehyde addition reaction during the gelation process*,* i.e., there is a certain amount of un-reacted monomers, leading to an incomplete gelation, and, as a consequence, to a material with a lower structural order. Other authors have also observed this phenomenon [47,48,49]. Podyacheva et al. [50] published an excellent review reporting different studies concerned with the creation of a higher number of defects on N-doped carbon materials, being these proportional to the doping extent.

The amount of nitrogen in CXGs was determined by elemental analysis. Table 2 shows the weight percentage of C, N, and H (sulphur content was below the detection limit in all cases). Pristine materials present a small amount of N, below 0.35 wt %, most probably coming from impurities in the carbon precursors. With the use of melamine, weight percentages of nitrogen around 3 wt % are introduced in the carbon xerogel. A slightly higher N content is observed for the N-CXG-300.

**Figure 2:**
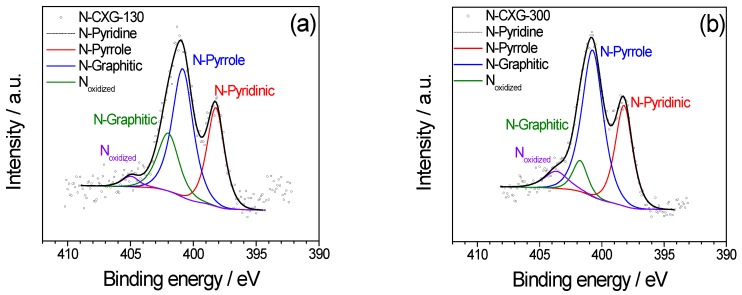
XPS spectra for N1s orbital for (**a**) N-CXG-130 and (**b**) N-CXG-300 xerogels.

The nature of the nitrogen functionalities for N-CXGs was investigated by X-ray photoelectron spectroscopy (XPS). N1s spectra were deconvoluted, as described in [30] into four components, as shown in Figure 2, considering the binding energy values detailed in Table 3.

Table 3 shows the N content, as well as the chemical speciation obtained by XPS. N-CXG-300 possesses a larger amount of N on the surface, in line with elemental analysis. It is believed that N atoms are distributed outside of the primary particles constituting the organic gel, as determined by Pérez-Cadenas et al. [40]. As gelation occurs in an aqueous media, the hydrophilic amine groups derived from the polymerization, become oriented to the external part of the water-solid interface of the primary particles. This appears to occur in a larger extent for higher R/C ratio, this is, lower than the amount of sodium carbonate (lower pH).

Doped carbon xerogels present comparable contents of pyridinic N, around 30 at %, being slightly more abundant for the N-CXG-300 taking into account the total amount of N (4.5 at %). Larger differences were found in the contribution of pyrrole, graphitic and oxidized N. While graphitic N content is substantially larger in N-CXG-130 (21.3% vs. 7.9%), N-CXG-300 has a greater concentration of both pyrrole and oxidized N. In fact, XPS spectra of Figure 2, show that the shape of the N1s peak is very similar for both materials, exhibiting two main peaks associated to pyridinic and pyrrolic nitrogen mostly, and differing in the signal at high binding energy (>401 eV), due to the variation in the presence of graphitic and oxidized N.

### 2.2. Pt Catalysts Supported on N-Doped Carbon Xerogels

**Figure 3:**
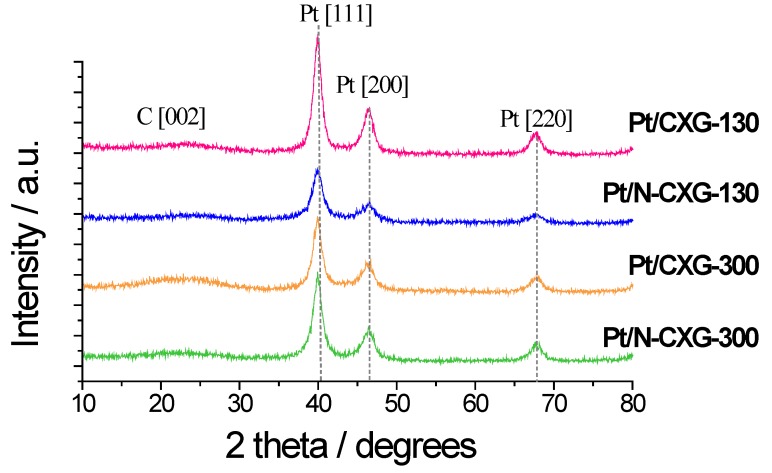
XRD patterns for Pt-catalysts.

Pt catalysts supported on CXGs (20 wt % Pt) were synthesized using formic acid as a reducing agent, as detailed in the experimental section. The X-ray diffraction (XRD) patterns (Figure 3) exhibited the typical face-centered cubic structure of Pt. Catalysts were compared to an internal benchmark, Pt supported on carbon black Vulcan (obtained by the same method). Pt crystal sizes, determined from XRD patterns shown in Figure 3, range between 3.4 nm and 5.5 nm. Pt content, shown in Table 4 along with Pt crystal sizes, approaches the nominal 20 wt %. This value is slightly lower for the catalysts supported on CXGs obtained with a R/C = 300.

Upon N-doping of the CXG support, Pt crystal size decreases for CXG-130 sample. It is well known that the variation of carbon support features, apart from nitrogen content, may also influence Pt particle size and distribution, such as the pore size and the surface area [51,52,53]. Nonetheless, the decrease of metal crystal size with N-doping of the support has been also described in the literature [54,55]. Some authors claim that nitrogen functionalities provide lone pairs of electrons in a sp^2^ orbital in the plane of the carbon ring. Being nitrogen sites less electronegative than oxygen-containing sites (predominating on carbon materials), Pt particles are more strongly anchored to pyridinic N sites, preventing their agglomeration [55]. Other authors determined that Pt atoms are confined in those sites where N replaces C, which is where Pt nanoparticle nucleation takes place, leading to a smaller crystal size [56]. This effect of N on crystallite size was not observed for the support characterized by higher R/C ratio, in which there is no significant variation.

**Figure 4:**
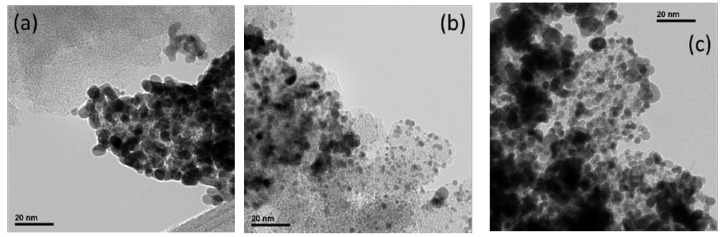
TEM micrographs for carbon xerogels (CXG)-supported Pt catalysts: (**a**) Pt/CXG-130; (**b**) Pt/N-CXG-130; and (**c**) Pt/N-CXG-300.

Figure 4 shows representative TEM micrographs for Pt/CXG catalysts. Pt/CXG-130, Figure 4a, presents some inhomogeneities in Pt particles distribution on the CXG-130 support, including Pt particle agglomerates and small uncatalyzed xerogel regions, due to the preferential growth of metal particles on support defects. The morphology of the CXG-130 xerogel is indeed very compact and dense, consistent with its microporous structure, as previously discussed regarding nitrogen physisorption measurements. Large microporosity, and thus low mesopores availability for Pt deposition, is most probably responsible for the observed poor metal dispersion. The catalyst Pt/N-CXG-130, shown in Figure 4b, presents a better Pt nanoparticle distribution on the xerogel surface, as a consequence of the support’s larger surface area and pores, with Pt particles of uniform size. Finally, the Pt/N-CXG-300 catalyst, as shown in Figure 4c, also showed well-distributed areas together with the presence of some agglomerations and clusters of particles.

### 2.3. Activity for the Oxygen Reduction Reaction

**Figure 5:**
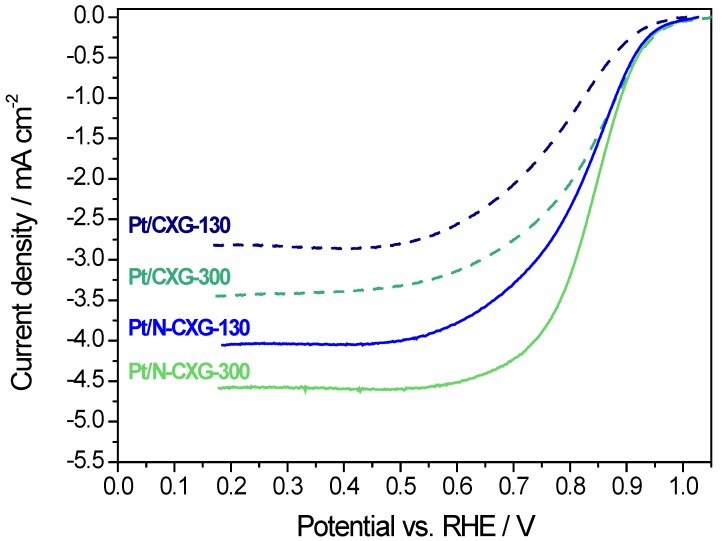
Linear sweep voltammetries obtained in a rotating disk electrode (RDE) in an O_2_-saturated 0.5 M H_2_SO_4_ solution at ω = 1600 rpm; scan rate: 5 mV·s^−1^.

The electrocatalytic activity of CXG-supported Pt catalysts was evaluated in a half-cell fitted with a rotating disk electrode (RDE). Catalysts were deposited as a thin film on the surface of the glassy carbon maintaining the Pt loading in all experiments (50 μg·cm^−2^), see details in the experimental section. Figure 5 shows the linear sweep voltammetries (LSV) obtained at 1600 rpm in an O_2_-saturated 0.5 M H_2_SO_4_ solution. The positive effect of N-doping is clearly evidenced in the figure. Both Pt/N-CXGs catalysts present better onset and half-wave potentials than their respective undoped counterparts. Moreover, better catalytic behavior is also accompanied by a higher limiting current density, indicating a reduction process approaching 4e- pathway. Among Pt/N-CXGs catalysts, the one supported on N-CXG-300 is more active for the oxygen reduction reaction (ORR) in terms of both limiting current density and onset potential.

Table 5 summarizes several parameters obtained from the RDE measurements along with the number of electrons exchanged (applying the Koutecky-Levich method) in the oxygen reduction reaction, as well as the electrochemical surface area (ECSA) calculated from the adsorption of hydrogen as determined by cyclic voltammetry in the deaerated base electrolyte. There is not a direct correlation between ECSA and electrocatalytic activity, suggesting that the variation in ORR activity is related to the intrinsic activity rather than to the dispersion of Pt. In this context, half-wave potential is a good indicator of the intrinsic activity in the mixed controlled zone (similar contributions from kinetics and diffusion phenomena), and this is not related to the available active sites (ECSA), but to the presence of nitrogen in the carbon xerogel. Indeed, the best results in terms of both half-wave potential and number of electrons were obtained for the nitrogen-doped CXG-supported catalysts, similar to our internal reference based on Vulcan carbon black support (Pt/Vulcan). On the other hand, the Pt catalysts supported on the un-doped carbon xerogels resulted in an inefficient reduction mechanism towards 2 e^−^ pathway, as ascertained from the values summarized in Table 5 of 2.3 and 2.8 for Pt/CXG-130 and Pt/CXG-300. This could be ascribed to a worse electron transfer from the support to the active phase, or to the higher extent of agglomeration of metal particles as envisaged from TEM images.

In the literature, the effect of different nitrogen moieties on doped carbon-supported electrocatalysts is not yet clear. Some recent studies claim an electronic interaction between nitrogen functionalities and platinum, affecting the surface electronic structure of the latter and modifying the adsorption/desorption of oxygen species [57,58]. In our case, the ORR electrochemical activity results evidenced that, the use of N-doped carbon material helps enhancing the activity of the Pt-catalyst, and, this enhancement is more acute when the N-doped CXG possesses a higher amount of N in the form of pyridine and pyrrole. Besides, N-CXG-300 shows a higher ratio of N-pyridine/N-graphitic (3.9 vs. 1.4 for N-CXG-130). Melke et al. observed that platinum-support interactions in N-doped carbon materials might influence several parameters involved in the oxygen reduction, such as Pt particle size, higher electron density of the support with nitrogen, and the prevention of the formation of O-type functional groups [59]. Therefore, even if the effect of N-doping is still under debate, it appears that a larger amount of pyridinic nitrogen favors the electrocatalytic activity of Pt.

**Figure 6:**
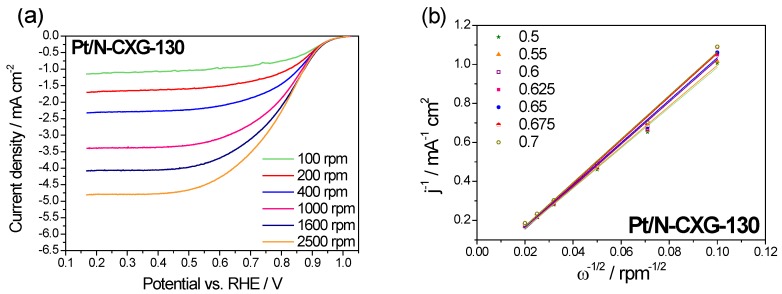
(**a**) LSV curves for Pt/N-CXG-130 at different rotation rates recorded in a 0.5 M H_2_SO_4_ O_2_-saturated solution. Scan rate: 5 mV·s^−1^; (**b**) Koutecky-Levich plots for Pt/N-CXG-130 at different potentials. The legend refers to potential values (V vs. RHE).

**Figure 7:**
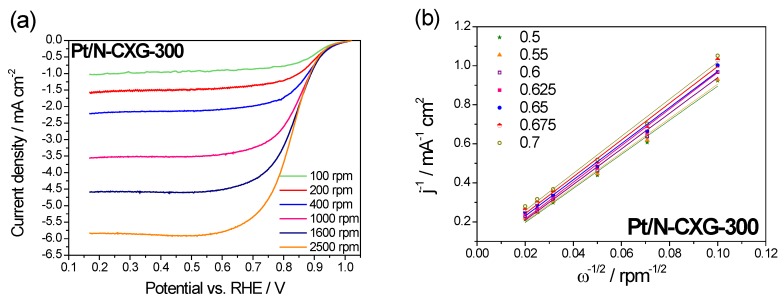
(**a**) Linear sweep voltammetries (LSV) curves for Pt/N-CXG-300 at different rotation rates recorded in a 0.5 M H_2_SO_4_ O_2_-saturated solution. Scan rate: 5 mV·s^−1^; (**b**) Koutecky-Levich plots for Pt/N-CXG-300 at different potentials. The legend refers to potential values (V vs. RHE).

The ORR catalytic pathway for Pt/N-CXGs catalysts was investigated by Koutecky–Levich (K-L) plots obtained from the LSV at various rotating speeds (100–2500 rpm), shown in Figure 6 and Figure 7. Figure 6a and Figure 7a show how the cathodic current increases when increasing the rotating speed. This is due to an improved oxygen mass transport at the electrode surface.

The Koutecky-Levich equation was employed to calculate the number of electrons transferred per O_2_ molecule in the ORR from the slopes of the plots shown in Figure 6b and Figure 7b, Pt/N-CXG-130 had a value of n of 3.5 e^−^ and Pt/N-CXG-300 had a n value of 3.8 e^−^. Other studies in the literature have determined that N-doped materials with a higher percentage of graphitic N usually are more active towards a 2 e^−^ pathway, resulting in a partial reduction of oxygen to hydrogen peroxide, whereas those with higher contents of pyridinic and pyrrolic N lead to direct reduction of O_2_ to water via a 4 e^−^ mechanism, as corroborated by our results. For example, Kurungot et al. stated that pyrrole is essential for oxygen adsorption and subsequent reduction to water via a 4e^−^ mechanism. Whereas, Ruoff et al. came to the conclusion that pyridinic nitrogen improves offset potential and converts mechanism from 2e^−^ to 4e^−^ while activity depends on the amount of graphitic nitrogen [60].

**Figure 8:**
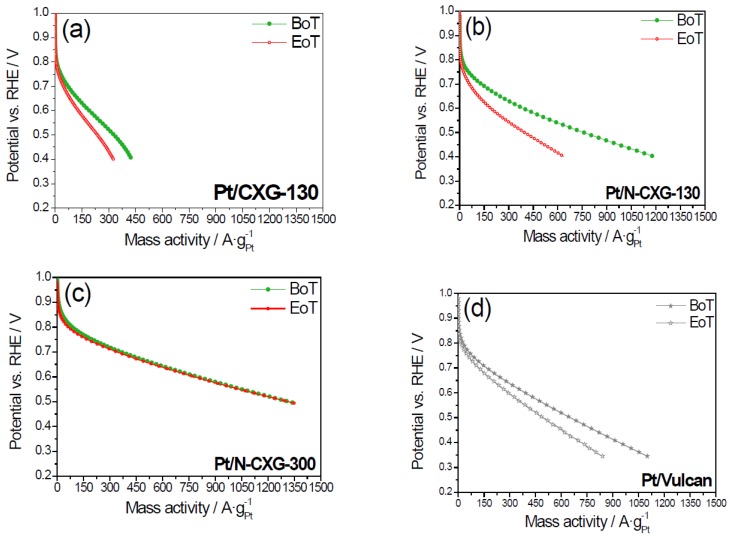
Linear sweep voltammetries obtained in a gas diffusion electrode (GDE) feeding O_2_ to the backing layer of the electrode at the beginning of test (BoT) and at the end of test (EoT), consisting of 1000 cycles between 0.6 and 1.2 V vs. RHE. 0.5 M H_2_SO_4_ solution; scan rate: 5 mV·s^−1^; 0.1 mg Pt cm^−2^.

Pt/N-CXGs were also investigated in a gas diffusion electrode (GDE) in a half-cell configuration and a low Pt loading (0.1 mg·cm^−2^) in order to assess their stability and performance under conditions more similar to a practical application. The catalysts were subjected to a 1000 cycles between 0.6 and 1.2 V vs. RHE of potential. Figure 6 shows the polarization curves at the beginning and at the end of the stress test (BoT and EoT, respectively). As evidenced in Figure 8a,b, the catalyst based on the N-doped CXG (Pt/N-CXG-130) presents a decay of performance of about 45%, which is larger that the decay obtained with its un-doped counterpart, of about 30% (Pt/CXG-130). These catalysts present similar physico-chemical features, i.e., similar support BET surface area, a parameter that greatly influences the resistance towards degradation. On the other hand, Pt/N-CXG-130 presents a lower Pt crystal size (4.3 nm vs. 5.9 nm for the Pt/CXG-130), which may also contribute to its lower stability [32]. On the other hand, Pt/N-CXG-300 (Figure 8c) presents almost the same performance at the beginning and at the end of the 1000 cycles. Compared with the Pt/N-CXG-130, presenting a higher decay of performance, this catalyst is supported on N-CXG-130, with a higher specific surface area compared to the N-CXG-300 support (500 m^2^·g^−1^ vs. 390 m^2^·g^−1^). The higher the surface area the faster the oxidation of carbon, which may have led to a higher corrosion of the CXG-130, and N-CXG-130 supported catalysts but it does not explain itself the excellent stability for Pt/N-CXG-300.

In the literature, the reason why catalysts supported on N-doped materials present higher stability is still not clear. Some authors refer that it is due to a better chemical binding between the support and the noble metal particles, which results in an enhanced durability [61,62]. In the present case, only the catalyst supported on the N-CXG-300 shows a remarkable stability. Pt/Vulcan suffers a significant decay of performance, but less acute than Pt/N-CXG-130. It is well-documented that carbon supports with a higher graphitic content are more corrosion resistant, while excess oxygen groups can lead to an acceleration of the carbon support corrosion rate [63]. Vulcan is characterized by a low Brunauer-Emmet-Teller (BET) surface area (250 m^2^·g^−1^) when compared to CXGs (≈380–500 m^2^·g^−1^). Besides, the lower the crystal size, the higher the corrosion rate. The remarkable durability of Pt/N-CXG-300 might be due to a combination of low surface area and appropriate nitrogen speciation on the surface, presumably within mesopores. As a consequence, the density of pyrrolic and pyridinic nitrogen groups per surface area is much higher, allowing Pt nanoparticles for an improved anchorage, thus hindering the coarsening of the active phase by the dissolution and redeposition of the noble metal particles (Ostwald ripening mechanism). Computational studies that will be the focus of a future work, will help to determine if the nature of N groups has any influence on the durability.

In order to better identify the causes of catalyst degradation, the ECSA was calculated from the hydrogen adsorption in the deaerated electrolyte by means of cyclic voltammetry before and after the degradation tests (Table 6).

Pt/N-CXG-300 suffers the lowest decay of ECSA, which is comparable to that of Pt/Vulcan. Whereas, Pt/Vulcan showed a decrease of current density that is proportional to the decay of ECSA, the Pt/N-CXG-300 showed a negligible variation of current. This means that the intrinsic activity in terms of current per unit of Pt surface area increased after the degradation test, balancing the loss of ECSA. On the other hand, Pt/N-CXG-130 and its un-doped counterpart, lost almost half of their ECSA, directly impacting the performance. Thus, using a carbon xerogel with well-defined porosity and nitrogen functionalities appears as a good strategy to improve the degradation resistance of Pt catalysts despite the decrease of electrochemical surface area and the loss of Pt active sites availability.

## 3. Materials and Methods

### 3.1. Synthesis of Materials

N-doped carbon xerogels were synthesized, as described by Gorgulho et al. [30]. Melamine (M), a nitrogen-precursor, resorcinol (R), sodium carbonate (C), and formaldehyde (F) were mixed in different proportions to obtain two different N-doped carbon xerogels. The resorcinol to sodium carbonate ratio was whether R/C = 130 or R/C = 300, resorcinol to formaldehyde ratio was maintained constant R/F = 0.5, as well as the R/M molar ratio, R/M = 5. Briefly, 9.09 g of R, 2 g of M and C were mixed in distilled water under stirring, temperature was risen up to 90 °C until the solution became transparent-yellow and subsequently cooled down to room temperature. F was subsequently added under continuous stirring for 30 min. The mixture was then poured into closed vials that were placed in an oven at 85 °C for 72 h to carry out the gelation. The gels were dried for 5 h at 65 °C, and then at 110 °C for another 5 h in a ventilated oven. The organic xerogels were pyrolyzed in a tubular reactor with flowing N_2_ for a total of 6 h in the following order: 150 °C for 2 h; 300 °C for 1 h; 600 °C for 1 h; and, 800 °C for 2 h. Carbon xerogels without melamine were also synthesized under the same synthesis conditions previously detailed excluding melamine, in order to study the influence of N-doping. Un-doped xerogels will be named as CXG with their corresponding R/C ratio (i.e., CXG-130), whereas an N will be added when talking about N-doped xerogels, i.e., N-CXG-130.

Catalysts with a 20 wt % of Pt loading were obtained using both bare (CXGs) and N-doped carbon xerogels (N-CXGs) as support. The synthesis method consisted of an impregnation and reduction with formic acid [64,65]. A 2 M solution of formic acid (Panreac) was pre-heated at 80 °C. Then, the carbon material was dispersed in this solution under stirring. Subsequently, an aqueous solution of the metallic precursor, H_2_PtCl_6_ (Sigma-Aldrich, Madrid, Spain) in 4 mM concentration, was added dropwise and stirred for 1 h at 80 °C. Once the mixture cooled down, it was filtered and washed with deionized water. The obtained powder was dried overnight.

### 3.2. Physico-Chemical Characterization

N_2_ physisorption at −196 °C (Micromeritics ASAP 2020) was employed to investigate the textural and morphological features of carbon xerogels. Different parameters were calculated from adsorption-desorption isotherms by applying different equations/methods: specific surface area (Brunauer-Emmet-Teller (BET) equation), pore volume (single-point method), and average pore size (Barrett-Joyner-Halenda (BJH) methods). XPS analyses were performed in a ESCA Plus Omicron spectrometer (Scienta Omicron, Uppsala, Sweden) equipped with a Mg (1253.6 eV) anode, 150 W (15 mA, 10 kV) power, over an area of sample of 1.75 mm × 2.75 mm. C1s, N1s, and Pt4f signals were obtained at 0.1 eV step, 0.5 s dwell, and 20 eV pass energy. Data analysis and quantification were performed using CasaXPS software (Casa Software Ltd, CasaXPS Version 2.3.18, Teignmouth, United Kingdom). A Shirley background subtraction was used for quantification of C1s, N1s, and Pt4f spectra. A 70% Gaussian/30% Lorentzian line shape was utilized in the curve deconvolution of spectra. Elemental analysis was performed with a Thermo Flash 1112 analyzer (Thermoscientific Waltham, MA, USA) for the determination of C, H, N, and S, in the range 0.05%–99.95%.

A Bruker AXS D8 Advance diffractometer (Bruker Española S.A, Madrid, Spain), with a θ-θ configuration and using Cu-Kα radiation, was employed to perform XRD analyses. Scherrer’s equation was applied to the (2 2 0) reflection of platinum to calculate the crystallite size. TEM images were obtained to evaluate the size and morphology of Pt particles. The microscope was a JEOL 2100F (JEOL USA, Inc., Peabody, MA, USA), equipped with a field emission electron gun with an accelerating voltage of 200 kV and a point resolution of 0.19 nm. 3 mg of the sample were dispersed in ethanol in an ultrasonic bath and then placed in a Cu carbon grid until the liquid phase evaporated.

### 3.3. Electro-Chemical Characterization

The electrochemical activity of Pt-catalysts was assessed both in rotating disk (RDE) and gas diffusion (GDE) working electrodes. In the first case, an ink consisting of a dispersion of the catalyst was deposited on a graphite rod of 5 mm diameter for a Pt loading of 50 μg·cm^−2^. In the second case, GDEs were prepared as described elsewhere [20]. A typical GDE consisted of the catalytic layer with 0.1 mg·cm^−2^ (±0.02 mg·cm^−2^) of Pt loading, deposited onto a hydrophobic backing layer (LT 1200W ELAT, E-TEK) and a gas diffusion layer. In both electrodes, RDE and GDE, the composition of the catalytic ink was 67 wt % catalyst and 33 wt % Nafion^®^ ionomer (DuPont, Wilmington, DE, USA).

A thermostated three-electrode half-cell connected to an Autolab Metrohm potentionstat/galvanostat (Methrom, Utrecht, the Netherlands) was employed to perform all of the electrochemical measurements. The reference electrode was a mercury-mercurous sulfate (Hg/Hg_2_SO_4_, sat. K_2_SO_4_) and the counter electrode was a high surface graphite rod. As electrolyte, a 0.5 M H_2_SO_4_ aqueous solution was employed. The activity towards the ORR was determined by performing linear sweep voltammetries at 25 °C under a continuous flow of pure oxygen. Besides, an accelerated stress test was performed with the GDE [66], to evaluate the resistance to degradation of the catalysts. Under N_2_ flow, 1000 cycles were performed cycling potential between 0.6 and 1.2 V vs. RHE. Cyclic voltammetry (from 0.02 V to 1.2 V vs. RHE) in N_2_ and linear sweep voltammetry curves in O_2_ were recorded to evaluate the decay process.

## 4. Conclusions

Carbon xerogels have been doped with nitrogen by a simple methodology introducing melamine in the gelation process of the organic gel. The structural and chemical characteristics have been investigated, observing that for an adequate porosity and to favor the formation of certain nitrogen functionalities, a trade-off situation is needed by optimizing the resorcinol to carbonate ratio.

Pt catalysts with low Pt loading supported on nitrogen-doped carbon xerogels were also synthesized, and their activity was evaluated in acid solution for the electro-reduction of oxygen. The presence of N appears to favor the complete reduction mechanism of oxygen to water through a 4 e^−^ pathway. Pt/N-CXGs present a higher activity towards the ORR than the un-doped carbon-supported counterparts. Besides, providing adequate physico-chemical features, i.e., low surface area and high N groups density, doping with N allows obtaining highly durable Pt catalysts.

## Figures and Tables

**Table 1:** Textural properties (determined from N_2_ adsorption isotherms) and structural parameters (as determined from Raman spectra).

Carbon Xerogel	S_BET_ (m^2^·g^−1^)	V_pore_ (cm^3^·g^−1^)	V_micro_ (cm^3^·g^−1^)	V_meso_ c(m^3^·g^−1^)	d_pore_ (nm)	I_D_/I_G_	G Band Position (cm^−1^)
CXG-130	461	0.29	0.23	0.06	3.6	0.74	1593.5
CXG-300	587	0.65	0.10	0.55	5.2	0.89	1591.3
N-CXG-130	497	1.35	0.14	1.21	19.2	0.98	1597.0
N-CXG-300	387	0.34	0.17	0.17	7.3	0.91	1595.4

**Table 2:** Weight percentage of C, N, and H determined by elemental analysis.

Carbon Xerogel	C (wt %)	H (wt %)	N (wt %)
CXG-130	94.93	1.08	0.11
CXG-300	94.58	0.84	0.35
N-CXG-130	90.12	0.92	3.0
N-CXG-300	93.30	0.82	3.4

**Table 3:** Nitrogen content and the different species deconvoluted from the X-ray photoelectron spectroscopy (XPS) N1s band.

Carbon Xerogel	N (at %)	N-Pyridine (at %)	N-Pyrrole (at %)	N-Graphitic (at %)	N_oxidized_ (at %)
		398.2 eV	400.8 eV	402.0 eV	405.0 eV
N-CXG-130	3.4	29.7	46.0	21.3	3.0
N-CXG-300	4.5	30.8	54.4	7.9	6.9

**Table 4:** Pt crystal size determined by XRD and percentage of Pt determined by TGA.

Catalyst	Pt Crystal Size (nm)	Pt Content (wt %)
Pt/CXG-130	5.9	20.7
Pt/CXG-300	5.4	15.0
Pt/N-CXG-130	4.3	18.9
Pt/N-CXG-300	5.5	16.7
Pt/Vulcan	3.4	16.7

**Table 5:** Kinetic parameters obtained from RDE measurements (potential vs. RHE), along with the electrochemical surface area (ECSA) calculated from cyclic voltammetry.

Catalyst	ORR Onset Potential (V vs. RHE) at −0.1 mA·cm^−2^	ORR Half-Wave Potential (V vs. RHE)	Limiting Current Density (mA·cm^−2^)	n (Koutecky-Levich)	ECSA (m^2^·g^−1^ Pt)
Pt/CXG-130	0.94	0.78	2.8	2.3	25
Pt/CXG-300	0.97	0.83	3.5	2.8	39
Pt/N-CXG-130	0.97	0.82	4.6	3.5	33
Pt/N-CXG-300	0.97	0.84	4.1	3.8	24
Pt/Vulcan	0.95	0.84	4.9	4.0	42

**Table 6:** Electrochemical surface area (ECSA) at the beginning and at the end of the accelerated degradation tests, calculated from cyclic voltammetry.

Catalyst	ECSA BoT (m^2^·g^−1^ Pt)	ECSA EoT (m^2^·g^−1^ Pt)	% Loss
Pt/CXG-130	25	14	47
Pt/N-CXG-130	33	19	42
Pt/N-CXG-300	24	16	33
Pt/Vulcan	42	28	33
